# Quality of Life Among Patients with Hematologic Malignancies Receiving Outpatient Care: Associations with Socioeconomic, Clinical, Psychological, and Physical Factors

**DOI:** 10.3390/jcm15218545

**Published:** 2026-11-03

**Authors:** Małgorzata Pudełek, Jarosław Dybko, Sebastian Zdończyk, Iwona Malicka

**Affiliations:** 1 Lower Silesian Oncology, Pulmonology and Hematology Center, 53-439 Wrocław, Poland; jaroslaw.dybko@dcopih.pl; 2 Department of Psychology, Pomeranian University in Słupsk, 76-200 Słupsk, Poland; sebastian.zdonczyk@upsl.edu.pl; 3 Department of Hematology and Bone Marrow Transplantation, Janusz Korczak Regional Specialist Hospital in Słupsk Ltd., 76-200 Słupsk, Poland; 4 Faculty of Physiotherapy, Wrocław University of Health and Sport Sciences, 51-612 Wrocław, Poland; iwona.malicka@awf.wroc.pl

**Keywords:** Hematologic malignancies, outpatient treatment, quality of life, rehabilitation

## Abstract

**Background:** Although therapeutic advances have prolonged survival in hematologic malignancies, these conditions may still significantly impair patients’ functioning and health-related quality of life (HRQoL). **Objective:** To assess HRQoL and selected socioeconomic, psychological, and physical factors in patients with hematologic malignancies receiving outpatient care. **Materials and Methods:** This study included 94 patients: 33 with chronic lymphocytic leukemia, 30 with plasma cell myeloma, and 31 with follicular lymphoma. The mean age of participants was 61.5 ± 13.2 years. HRQoL was measured using the European Organisation for Research and Treatment of Cancer Quality of Life Questionnaire-C30 version 3.0 (EORTC QLQ-C30); anxiety was evaluated using the State-Trait Anxiety Inventory (STAI); psychological adjustment to cancer was assessed using the Mini-Mental Adjustment to Cancer (Mini-MAC) scale; and dominant-hand grip strength was recorded. Differences between groups and factors associated with global health status/quality of life were analyzed. **Results:** The median global health status/quality-of-life score (QL2) was 66.66 (IQR: 25.00) in patients with chronic lymphocytic leukemia, 58.33 (IQR: 16.67) in patients with multiple myeloma, and 58.33 (IQR: 16.67) in patients with follicular lymphoma, on a 0–100 scale. Differences among diagnostic groups did not reach the predefined threshold for statistical significance (H(2, *N* = 94) = 5.7; *p* = 0.057). The median state anxiety score was 35.50 points (IQR:12), and the median trait anxiety score was 44.50 points (IQR:14). Significant differences between diagnoses were identified for trait anxiety (H(2, *N* = 94) = 16.76; *p* = 0.0002), whereas no statistically significant differences were observed for state anxiety (H(2, *N* = 94) = 5.19; *p* = 0.070). Moderate levels of constructive coping (median 44.00 points, IQR:5) and low levels of destructive coping (median 24.50 points, IQR:11) were observed, indicating a predominance of adaptive coping strategies; no significant between-group differences were detected. The median dominant-hand grip strength was 28 kg (IQR:18 kg), with no significant differences between diagnoses. Regression analysis showed that financial difficulties, trait anxiety, and dominant-hand grip strength were independently associated with the QL2 score (R^2^ = 0.431; *p* < 0.001). **Conclusions:** Financial difficulties, trait anxiety, and dominant-hand grip strength were independently associated with global health status/quality of life in patients with hematologic malignancies receiving outpatient care, although the cross-sectional design precludes causal conclusions.

## 1. Introduction

Hematologic malignancies comprise a heterogeneous group of neoplastic diseases, primarily encompassing leukemias, lymphomas, and myeloma. Alongside demographic changes, the burden of these diseases has risen, particularly among older adults, reflecting population aging, increased life expectancy, and advances in diagnostic and therapeutic approaches [1,2,3].

Hematologic malignancies are characterized by a heterogeneous clinical course, the use of intensive and often multistage treatment strategies, and the possibility of disease relapse, all of which impose a substantial burden on patients [4]. Symptoms arising from the malignancy itself, as well as adverse effects of treatment, may result in impaired physical functioning, reduced daily activity, and psychological and social challenges. Commonly reported symptoms include anemia, fatigue, cognitive impairment, including difficulties with concentration and memory, musculoskeletal pain, muscle cramps, and symptoms of anxiety and depression [5,6,7].

Contemporary approaches to cancer treatment focus not only on prolonging survival but also on maintaining optimal quality of life. These approaches emphasize the importance of evaluating treatment outcomes from the patient’s perspective [8,9,10]. Health-related quality of life (HRQoL) is particularly important in this context, as it provides a comprehensive assessment of how disease and treatment affect patients’ daily functioning.

HRQoL is a multidimensional concept encompassing physical, psychological, and social aspects of health, assessed within the context of patients’ individual experiences, expectations, and perceptions [11]. In individuals with hematologic malignancies, HRQoL assessment is particularly important due to the extended duration of treatment, persistent disease-related symptoms, and treatment toxicity, which may continue even during remission periods [12,13]. Although recent research has increasingly addressed the needs of inpatients, especially those undergoing intensive anticancer therapies and experiencing related complications, the experiences of outpatients remain underexplored. Outpatients, despite receiving less intensive direct medical supervision, often require prolonged treatment, regular health monitoring, and self-management of disease symptoms and treatment-related adverse effects at home. These factors can substantially affect their physical, psychological, and social functioning, as well as their HRQoL. The limited number of studies on this population highlights the need to evaluate their experiences further and identify factors affecting functioning and well-being during outpatient care. Therefore, this study aimed to assess HRQoL and examine the associations between selected socioeconomic, clinical, psychological, and physical factors and HRQoL in patients with hematologic malignancies receiving outpatient care.

## 2. Materials and Methods

### 2.1. Participants and Recruitment

This single-center observational study was conducted at the Hematology and Transplant Center of the Lower Silesian Oncology, Pulmonology, and Hematology Center in Wrocław, Poland. Participant recruitment was conducted from 15 January 2024 to 30 May 2025. Consecutive patients who met the inclusion criteria during the recruitment period were eligible for inclusion in the study. A total of 99 patients with hematologic malignancies receiving outpatient care were recruited. Of these, 94 patients provided complete questionnaire data and were included in the final study population: 33 with chronic lymphocytic leukemia, 30 with plasma cell myeloma, and 31 with follicular lymphoma. Treatment approaches differed across the diagnostic groups. Patients with chronic lymphocytic leukemia received venetoclax in combination with an anti-CD20 monoclonal antibody (obinutuzumab or rituximab), venetoclax monotherapy, or observation without active treatment. Patients with plasma cell myeloma received daratumumab monotherapy, daratumumab in combination with bortezomib, daratumumab plus lenalidomide, lenalidomide monotherapy, teclistamab, or carfilzomib. Patients with follicular lymphoma received O-CHOP (obinutuzumab, cyclophosphamide, doxorubicin, vincristine, and prednisone) or R-CHOP (rituximab, cyclophosphamide, doxorubicin, vincristine, and prednisone). The mean age of the participants was 61.5 ± 13.2 years, mean height was 169.73 ± 9.26 cm, mean body weight was 77.15 ± 16.26 kg, and mean body mass index (BMI) was 26.62 ± 4.43 kg/m^2^.

Inclusion criteria included age greater than 18 years, a confirmed diagnosis of hematologic malignancy, eligibility for outpatient treatment with monoclonal antibodies as monotherapy or in combination with immunochemotherapy, and written informed consent for study participation.

Exclusion criteria included intellectual or physical disabilities that prevented participants from completing the study procedures and absence of informed consent to participate in the study.

### 2.2. Research Methods

Participants’ quality of life was evaluated using the European Organisation for Research and Treatment of Cancer Quality of Life Questionnaire-C30 version 3.0 (EORTC QLQ-C30). Anxiety was assessed with the State-Trait Anxiety Inventory (STAI-X1 for state anxiety and X2 for trait anxiety). Psychological adjustment to cancer was measured using the Mini-Mental Adjustment to Cancer Scale (Mini-MAC), and dominant-hand grip strength was also recorded.

#### 2.2.1. Health-Related Quality of Life

HRQoL was assessed using the standardized EORTC QLQ-C30 version 3.0 [14]. This questionnaire consists of 30 items that generate a multidimensional quality-of-life profile and is structured into three principal components:

1. Global health status and quality-of-life scale (QL2)—questions 29 and 30.
2. Functional scales:
  2.1. Physical functioning scale (PF2): questions 1 to 5.
  2.2. Role functioning scale (RF2): questions 6 and 7.
  2.3. Emotional functioning scale (EF): questions 21 to 24.
  2.4. Cognitive functioning scale (CF): questions 20 and 25.
  2.5. Social functioning scale (SF): questions 26 and 27.
3. Symptom scales:
  3.1. Fatigue scale (FA): questions 10, 12, and 18.
  3.2. Nausea and vomiting scale (NV): questions 14 and 15.
  3.3. Pain scale (PA): questions 9 and 19.
  3.4. Dyspnea scale (DY): question 8.
  3.5. Insomnia scale (SL): question 11.
  3.6. Appetite loss scale (AP): question 13.
  3.7. Constipation scale (CO): question 16.
  3.8. Diarrhea scale (DI): question 17.
  3.9. Financial difficulties scale (FI): question 28.

Items 1 to 28 are assessed using a four-point response scale ranging from 1 (“not at all”) to 4 (“very much”), whereas items 29 and 30, which assess global health status and quality of life, use a seven-point scale ranging from 1 (“very poor”) to 7 (“excellent”) [15,16].

Scores for individual scales are transformed according to the standard EORTC procedure to a range of 0 to 100 points.

Score interpretation depends on the scale type. On functional scales and QL2, higher scores reflect better functioning or higher quality of life. On symptom scales, higher scores represent increased symptom severity or more significant problems, indicating poorer quality of life within the respective domain.

The EORTC QLQ-C30 provides a comprehensive assessment of patients’ quality of life, accounting for both functioning across domains and the burden of cancer and treatment-related symptoms.

The EORTC QLQ-C30 demonstrates robust psychometric properties. Cronbach’s α coefficients for its multi-item scales typically exceed 0.70 in validation studies, and test–retest reliability ranges from 0.80 to 0.90 [17,18].

#### 2.2.2. State Trait Anxiety Inventory

Anxiety was assessed using the STAI, originally developed by C.D. Spielberger, R.L. Gorsuch, and R.E. Lushene [19], and adapted into Polish by J. Strelau, T. Tysarczyk, and K. Wrześniewski [20]. This instrument measures two relatively independent dimensions of anxiety: STAI-X1 and STAI-X2, with each scale comprising 20 statements.

The first section of the (X1) STAI assesses anxiety as a current emotional state. This component is highly sensitive and allows for the detection of changes in anxiety over short periods. The second section (X2) evaluates anxiety as a relatively stable personality trait. Each item is rated using a four-point response scale. Scores for both subscales are calculated by summing the responses to individual items, with reverse-scored items accounted for. Each scale yields a score ranging from 20 to 80 points, with higher scores reflecting greater levels of anxiety.

STAI-X1 and STAI-X2 scores are interpreted separately, which enables differentiation between current and situational anxiety and a relatively stable predisposition toward anxiety.

The STAI has established psychometric properties. The Polish adaptation demonstrates high internal consistency for both subscales, with Cronbach’s α coefficients ranging from 0.89 to 0.92 for X1 and from 0.76 to 0.90 for X2 in adult populations [20].

#### 2.2.3. Mini-Mental Adjustment to Cancer Scale

Psychological adjustment to cancer was assessed using the Mini-MAC, developed from the MAC questionnaire by Watson et al. [21] and adapted for use in Poland by Z. Juczyński [22]. This instrument assesses cognitive and emotional responses to a cancer diagnosis and identifies strategies employed by patients during the adjustment process.

The Polish version of the Mini-MAC comprises 29 statements and enables assessment of four adjustment strategies to cancer:

- Anxious preoccupation, which reflects anxiety related to the illness and the perception of the disease as disturbing, uncontrollable, and threatening.
- Fighting spirit, characterized by perceiving the illness as a challenge and actively coping through measures such as seeking alternative therapies or engaging in activities such as dancing, traveling, or physical activity.
- Helplessness–hopelessness, defined by feelings of being lost and powerless, which often lead to withdrawal from everyday life and cessation of occupational activities.
- Positive reappraisal or redefinition, which entails a shift in attitude towards life and an increased appreciation of its value in the face of progressive illness.

Anxious preoccupation and helplessness–hopelessness are components of the passive (destructive) coping style, while the remaining two dimensions constitute the active (constructive) coping style.

Respondents rate each Mini-MAC statement on a four-point scale, with 1 indicating “definitely not” and 4 indicating “definitely yes”. Scores for each coping strategy are calculated separately by summing relevant item scores, resulting in a possible total score ranging from 7 to 28 points. Higher scores reflect greater intensity of behaviors characteristic of the respective coping strategy [22].

The Polish version of the Mini-MAC demonstrates high reliability, with Cronbach’s α coefficients ranging from 0.87 to 0.92 for the individual adjustment strategies [22].

#### 2.2.4. Handgrip Strength

Muscle strength was evaluated by measuring handgrip strength (HGS) with a Jamar hand dynamometer (Patterson Medical, Warrenville, IL, USA). HGS represents the maximum force exerted during gripping. This measurement indicates overall physical and muscular status [23,24,25].

HGS measurements followed the standardized protocol recommended by the American Society of Hand Therapists (ASHT). Participants sat with their trunks upright, feet supported on the floor, shoulders in a neutral and adducted position, elbows flexed at 90°, forearms in a neutral position, and wrists in a neutral position. Each hand was measured using a maximal brief grip of the dynamometer. The highest value from two consecutive trials was recorded for analysis and expressed in kilograms (kg) [26]. Only dominant-hand grip strength HGS was included in the analyses for this study.

HGS demonstrates high measurement reliability in patients with cancer (intraclass correlation [ICC] > 0.90) [27].

### 2.3. Ethics

Approval for the study was granted by the Senate Research Ethics Committee of Wrocław University of Health and Sport Sciences (approval no. 28/2023; approved on 12 January 2024).

### 2.4. Statistical Analysis

Statistical analyses were performed using Statistica version 13.1 (StatSoft, Inc., OK, USA). The normality of variable distributions was assessed using the Shapiro–Wilk test. As the analyzed data did not meet the assumption of normality, non-parametric methods were used. Data are presented as the median and interquartile range (IQR).

Differences among the three diagnostic groups were assessed using the Kruskal–Wallis test. To account for multiple testing across 24 outcomes, omnibus *p*-values were adjusted using the Benjamini–Hochberg false discovery rate (FDR) procedure, with the significance threshold set at q = 0.05. For outcomes with nominally significant omnibus tests, pairwise comparisons were performed using Dunn’s test with Holm adjustment for the three pairwise comparisons within each outcome. Both unadjusted and FDR-adjusted omnibus *p*-values are reported, and conclusions regarding overall between-group differences are based on FDR-adjusted *p*-values. Effect sizes for the Kruskal–Wallis tests were quantified using the epsilon-squared coefficient (ε^2^) and interpreted as follows: ε^2^ < 0.01, negligible effect; 0.01–0.06, small effect; 0.06–0.14, moderate effect; and ε^2^ ≥ 0.14, large effect [28].

Hierarchical linear regression analysis was performed to examine factors independently associated with global health status/quality of life (QL2), assessed using the EORTC QLQ-C30. Three hierarchical models were constructed, with successive blocks of explanatory variables added at each step. Model fit was assessed using the coefficient of determination (R^2^), adjusted R^2^, and the overall F-test. Regression coefficients were reported as unstandardized coefficients (B) and standardized coefficients (β), together with their *p*-values and 95% confidence intervals (CIs). The CIs for unstandardized coefficients were calculated using model-based standard errors and the t distribution with the appropriate residual degrees of freedom.

Regression assumptions and model diagnostics were assessed using variance inflation factors (VIFs) to evaluate multicollinearity; residual-versus-fitted plots and the Shapiro–Wilk test of residuals to assess residual patterns and normality; the Breusch–Pagan test to assess heteroscedasticity; and the Ramsey RESET test to evaluate model specification. Potentially influential observations were examined using Cook’s distance, leverage, and externally studentized residuals.

Internal model stability was assessed using 2000 pairs-bootstrap replications. In each replication, the prespecified 10-degree-of-freedom linear regression model was refitted without variable selection. Bootstrap percentile 95% confidence intervals for the unstandardized regression coefficients were calculated using the 2.5th and 97.5th percentiles of the 2000 bootstrap coefficient estimates. Model optimism in R^2^ was estimated by comparing model performance in each bootstrap sample with performance in the original dataset. The mean bootstrap optimism was subtracted from the apparent R^2^ to obtain the optimism-corrected R^2^. A fixed random seed (20261020) was used to ensure reproducibility.

Statistical significance was set at *p* < 0.05. For the between-group analyses, FDR-adjusted *p*-values were used to determine the significance of omnibus tests, whereas Holm-adjusted *p*-values were used to evaluate pairwise comparisons.

The minimum sample size was determined a priori using G*Power (version 3.1.9.7) through a power analysis for a linear multiple regression model, with a fixed model and R^2^ increase. Assuming a medium effect size (f^2^ = 0.15), a significance level of α = 0.05, and a statistical power of 0.80, the analysis indicated that at least 78 participants were required for the specified test. This calculation was based on the assumptions of the planned regression analysis and was not intended to establish adequate statistical power or precision for every coefficient in the final model. To account for the possibility of missing data, the planned sample size was increased by 15%, yielding a target sample size of 93 participants.

## 3. Results

The study groups demonstrated comparable demographic and sociodemographic characteristics. Regarding clinical characteristics, statistically significant differences between the groups were observed in the treatment approach and the distribution of treatment lines, whereas no statistically significant differences were found in the other clinical parameters. Table 1 provides detailed results.

Statistically significant differences in financial difficulties were observed between the study groups (H(2) = 9.03; *p* = 0.01). The mean ranks were 50.97 for patients with leukemia, 37.08 for patients with myeloma, and 53.89 for patients with lymphoma. Multiple-comparison analysis using the Dunn–Holm procedure revealed statistically significant differences between patients with leukemia and those with myeloma (*p* < 0.05), as well as between patients with myeloma and those with lymphoma (*p* < 0.05). The effect size (ε^2^ = 0.077) indicates a small effect (Table 2).

Statistically significant differences in trait anxiety were also observed between the study groups (H(2) = 16.77; *p* = 0.0002). The mean ranks were 31.84 for patients with leukemia, 55.80 for those with myeloma, and 56.13 for those with lymphoma. Multiple-comparison analysis using the Dunn–Holm procedure demonstrated that patients with leukemia had significantly lower STAI-X2 scores than patients with myeloma and lymphoma (*p* < 0.05). No statistically significant difference was observed between patients with myeloma and those with lymphoma (*p* > 0.05). The effect size (ε^2^ = 0.162) suggests a moderate effect (Table 2).

Statistically significant differences among the study groups were also observed for positive reappraisal/redefinition as a coping strategy (H(2) = 17.73; *p* = 0.0001). The mean ranks for positive reappraisal/redefinition were 31.53 for patients with leukemia, 55.61 for those with myeloma, and 56.64 for those with lymphoma. Multiple-comparison analysis using the Dunn–Holm procedure revealed that patients with leukemia had significantly lower positive reappraisal/redefinition scores than patients with myeloma and lymphoma (*p* < 0.05). No statistically significant difference was found between patients with myeloma and those with lymphoma (*p* > 0.05). The effect size (ε^2^ = 0.173) indicated a moderate effect (Table 2).

Following adjustment for multiple outcome testing, between-group differences remained significant for STAI-X2 and PR (both FDR-adjusted *p* = 0.003). The nominal difference in financial difficulties (unadjusted *p* = 0.010) did not remain significant after FDR adjustment (adjusted *p* = 0.087) and is now interpreted as an exploratory finding.

Detailed results for all assessed variables, including QLQ-C30, STAI, Mini-MAC, and dominant-hand grip strength, are provided in Table 2.

Hierarchical linear regression analysis was performed to examine factors independently associated with global health status (QL2), assessed using the EORTC QLQ-C30. The model was constructed a priori according to the conceptual ordering of the examined domains. Variables were entered in three sequential blocks: (1) demographic, socioeconomic, and clinical characteristics; (2) psychological factors; and (3) physical health and symptom-related factors. This approach allowed the contribution of each block to the explained variance in QL2 to be assessed.

Model 1 included demographic variables (age, sex), clinical variables (treatment duration, disease diagnosis), and financial difficulties. The model was statistically significant (F = 5.843, *p* < 0.001) and explained 28.9% of the variance in the outcome (R^2^ = 0.289; adjusted R^2^ = 0.240). Financial difficulties were significantly associated with QL2 (β = −0.491, *p* < 0.001), indicating that greater financial difficulties were associated with lower QL2 scores. Diagnosis was also significant: compared with patients with leukemia (the reference category), patients with myeloma had a lower QL2 score (β = −0.227, *p* = 0.038). The remaining variables did not reach statistical significance, although age showed a borderline association with QL2 (β = −0.177, *p* = 0.060).

Model 2 was expanded to include psychological variables, specifically trait anxiety (STAI-X2) and the constructive coping component of positive reappraisal or redefinition. The inclusion of these variables increased the explained variance by 9.7% (ΔR^2^ = 0.097). Model 2 was statistically significant (F = 6.628, *p* < 0.001) and explained 38.6% of the variance in the outcome (R^2^ = 0.386; adjusted R^2^ = 0.328). Within this model, financial difficulties (β = −0.404, *p* < 0.001) and STAI-X2 (β = −0.304, *p* = 0.002) remained significant predictors. After psychological variables were added to the model, age was also significantly associated with QL2 (β = −0.221, *p* = 0.024), while diagnosis was no longer statistically significant.

Model 3 incorporated additional physical health-related variables, specifically dominant-hand grip strength and fatigue. The inclusion of these predictors increased the explained variance by a further 4.5% (ΔR^2^ = 0.045). Model 3 remained statistically significant (F = 6.212, *p* < 0.001), accounting for 43.1% of the total variance in the outcome (R^2^ = 0.431; adjusted R^2^ = 0.361). In the final model, financial difficulties (β = −0.329, *p* = 0.001), trait anxiety (β = −0.248, *p* = 0.016), and dominant-hand grip strength (β = 0.284, *p* = 0.045) were independently associated with QL2. The remaining variables were not significant predictors in the final model.

After adjustment for all variables included in the final model, financial difficulties, trait anxiety, and dominant-hand grip strength remained independently associated with QL2. The association between diagnosis and QL2 observed in Model 1 was no longer statistically significant after psychological and physical health-related factors were included. Detailed results are presented in Table 3.

Regression diagnostics were performed to assess the adequacy of the models. These included variance inflation factors, inspection of residual-versus-fitted plots, the Shapiro–Wilk test of residuals, the Breusch–Pagan test for heteroscedasticity, the Ramsey RESET specification test, and influence diagnostics (Cook’s distance, leverage, and externally studentized residuals). Unstandardized regression coefficients are reported with 95% confidence intervals (Table 3).

Internal model stability was explored using 2000 pairs-bootstrap resamples. The apparent and adjusted R^2^ of the final model were 0.431 and 0.361, respectively. The estimated bootstrap optimism in R^2^ was 0.127, corresponding to an optimism-corrected R^2^ of approximately 0.304. Bootstrap percentile intervals were calculated for unstandardized coefficients (Supplementary Table S1). Given the sample size and single-center design, these estimates should be interpreted cautiously and externally validated.

Additionally, to assess possible multicollinearity related to handgrip strength, we calculated variance inflation factors (VIFs) for all predictors and fitted an intermediate regression model including HGS before adding fatigue. All models were estimated on the same complete-case sample. In the final model, the highest VIF was 2.82 (HGS), while the VIF for sex was 2.45, suggesting no severe multicollinearity. The standardized coefficient for sex changed from 0.021 (Model 2) to 0.210 (after adding HGS) and 0.243 (final model), but remained statistically nonsignificant (*p* = 0.067). This change should be interpreted in light of the strong association between sex and absolute HGS (Table 4).

A sensitivity analysis of the sample-size requirements indicated that approximately 118 participants would be needed to achieve 80% power for the overall regression model, assuming a medium effect size (f^2^ = 0.15), α = 0.05, and 10 predictor degrees of freedom. With 94 participants, the estimated power under these assumptions was approximately 0.67. These results suggest that the study may have had limited power to detect effects of this magnitude.

## 4. Discussion

Contemporary oncology considers health-related quality of life (HRQoL) a key treatment outcome alongside overall survival and progression-free survival [29,30]. In hematologic malignancies, therapeutic advances have substantially improved survival [31]; consequently, patients’ everyday functioning and well-being have gained increasing importance. A literature review indicates that quality of life is reduced among patients with hematologic malignancies across different diagnoses [32]. In the present study, the median global health status/quality-of-life score (QL2) was 66.66 (IQR: 25.00) in patients with chronic lymphocytic leukemia, 58.33 (IQR: 16.67) in patients with multiple myeloma, and 58.33 (IQR: 16.67) in patients with follicular lymphoma, on a 0–100 scale. Although the median scores were numerically lower in the multiple myeloma and follicular lymphoma groups than in the chronic lymphocytic leukemia group, the between-group differences did not reach statistical significance. The median scores observed in the three diagnostic groups were within the range reported in published EORTC QLQ-C30 reference data for cancer populations; however, such comparisons should be interpreted cautiously because reference values may vary according to patient and disease characteristics [33].

Quality of life in patients with hematologic malignancies is associated with multiple socioeconomic, clinical, psychological, and physical factors. The present study identified financial difficulties, trait anxiety, and dominant-hand grip strength as factors independently associated with the QL2 score in the final regression model. These findings suggest that both psychosocial and physical factors are relevant to quality of life in this patient population. Given the cross-sectional design, however, these associations should not be interpreted as causal relationships.

The importance of financial circumstances is supported by studies involving patients with cancer [34]. The costs and economic consequences associated with cancer treatment can place a substantial burden on patients and adversely affect their daily functioning. Financial burdens can negatively affect psychosocial well-being and contribute to a decline in quality of life. This issue persists even in countries with advanced, universally accessible healthcare systems, indicating that access to healthcare services does not fully mitigate the economic impact of cancer [35]. In oncology, the concept of financial toxicity encompasses both the objective financial burden associated with healthcare costs and the subjective distress experienced by patients in response to their financial circumstances [36,37,38]. In the present study, financial difficulties were assessed using a single item from the EORTC QLQ-C30. Although this item captures patients’ perceived financial difficulties, it is not a comprehensive measure of socioeconomic status or financial toxicity. The observed association with QL2 should therefore be interpreted in this context. Furthermore, because both measures were obtained using the same questionnaire, shared method variance may have contributed to the observed association.

Patients’ experiences and adaptation to the burdens associated with cancer may be related not only to their current clinical condition and financial situation but also to individual predispositions and psychological resources [39]. In the final regression model, higher trait anxiety was independently associated with lower QL2 scores. Higher levels of trait anxiety were observed in patients with multiple myeloma and follicular lymphoma compared with those with chronic lymphocytic leukemia. This finding is consistent with the literature documenting anxiety and depression among patients with hematologic malignancies, including patients with lymphoma and myeloma [40,41]. These differences may reflect a range of psychological and clinical factors. However, disease trajectory, concerns about relapse or progression, and other disease-specific characteristics were not directly assessed in this study. Therefore, these factors should be considered potential explanations rather than established determinants of the observed differences. Further research is needed to investigate the factors underlying differences in trait anxiety across diagnostic groups.

The diagnostic groups differed significantly in treatment approaches and the distribution of treatment lines, reflecting disease-specific therapeutic strategies. Patients were classified according to their treatment line or management approach, including active observation where applicable. These differences should be considered when interpreting between-group variations in anxiety and quality of life; however, the cross-sectional design precludes determining the extent to which these outcomes were associated with diagnosis or treatment-related factors.

At the same time, coping with the disease does not depend solely on the level of anxiety experienced but may also involve the interplay of multiple psychological factors, such as illness perception, sense of control, personal resources, and available stress-coping strategies [39,42]. The present study found that patients with multiple myeloma and follicular lymphoma reported greater use of positive reappraisal or redefinition than patients with chronic lymphocytic leukemia. However, constructive coping did not differ significantly between the diagnostic groups and was not independently associated with QL2 in the final regression model. Therefore, this finding should be interpreted cautiously and does not establish that patients in particular diagnostic groups adapt more successfully to their illness. Further research is needed to clarify the relationship between specific coping strategies and HRQoL in different hematologic malignancies.

Dominant-hand grip strength was positively associated with the QL2 score in the final regression model, indicating that greater grip strength was associated with better global health status/quality of life after adjustment for the other variables included in the model. Handgrip strength is an objective measure of muscle strength and may provide information about physical health in patients with cancer [43]. Reduced muscle strength may reflect the effects of cancer, ongoing treatment, and muscle loss associated with conditions such as sarcopenia and cancer cachexia [44]. Nevertheless, handgrip strength alone does not provide a comprehensive assessment of overall functional capacity, sarcopenia, or cachexia. Although reduced muscle strength may coexist with limitations in everyday activities and poorer physical well-being, the cross-sectional design does not allow the direction of this relationship to be established or the contribution of other clinical and personal factors to be excluded.

In summary, among patients with hematologic malignancies receiving outpatient care, including those undergoing treatment and those under observation, financial difficulties, trait anxiety, and dominant-hand grip strength were independently associated with global health status/quality of life. These findings highlight the relevance of socioeconomic, psychological, and physical factors in the assessment of HRQoL. A multidimensional assessment may help identify patients’ potential supportive-care needs alongside disease- and treatment-related considerations. However, the present observational study did not evaluate the effectiveness of any specific supportive-care intervention and therefore cannot establish that implementing a comprehensive, interdisciplinary care model would improve HRQoL.

### 4.1. Limitations

Several limitations should be acknowledged. The single-center design and relatively small sample size may limit the generalizability of the findings, while the cross-sectional design and lack of long-term follow-up preclude assessment of causal relationships and changes in quality of life over time.

Data on treatment response and treatment-related toxicities were not collected. Furthermore, although ECOG performance status and comorbidities were documented, they were not included in the regression models. Differences in disease course and treatment characteristics across diagnostic groups, together with the lack of detailed clinical data, may have resulted in residual confounding.

Financial difficulties were assessed using a single EORTC QLQ-C30 item, which is not a comprehensive measure of socioeconomic status or financial toxicity. As this item and the global health status/quality-of-life outcome (QL2) were derived from the same questionnaire, shared method variance may also have contributed to the observed association.

Finally, excluding patients unable to complete the study assessments may limit the applicability of the findings to those with greater cognitive or physical limitations. Future multicenter longitudinal studies with larger samples and more comprehensive clinical and treatment-related data are warranted to validate these findings.

### 4.2. Strengths

Key strengths of this study include the assessment of patients receiving outpatient treatment, which enabled analysis of quality of life under conditions resembling everyday functioning. The use of validated questionnaires ensured reliable assessment of quality of life. Additionally, the inclusion of socioeconomic, clinical, psychological, and functional variables enabled a multidimensional evaluation of factors influencing patients’ quality of life.

### 4.3. Future Research Directions

Future research should investigate how disease-related, psychological, socioeconomic, and physical factors jointly contribute to changes in quality of life over time. It should also evaluate whether targeted supportive interventions addressing anxiety, financial difficulties, and physical functioning are effective in improving patients’ quality of life.

## 5. Conclusions

Among patients with hematologic malignancies receiving outpatient care, financial difficulties, trait anxiety, and dominant-hand grip strength were independently associated with global health status/quality of life. These findings highlight the relevance of socioeconomic, psychological, and physical factors in the assessment of quality of life in this population. Further research is needed to confirm these associations and determine whether targeted supportive interventions can improve patients’ quality of life.

## Figures and Tables

**Table 1:** Characteristics of the study groups.

**Variable**	**Leukemia**	**Myeloma**	**Lymphoma**	***p*** **-Value**
*n*	33	30	31	
**Age** [years]; Median (IQR)	61.00; 22.00	66.00; 11.00	65.00; 20.00	0.348 ^a^
**BMI** [kg/m^2^]; Median (IQR)	25.83; 4.60	25.89; 6.20	26.70; 6.17	0.475 ^a^
**Gender—*n*; %**				
Female	14; 42.42%	18; 60.00%	14; 45.16%	0.331 ^b^
Male	19; 57.58%	12; 40.00%	17; 54.84%	
**Education—*n*; %**				
Primary	2; 6.06%	3; 10.34%	4; 12.90%	0.425 ^b^
Vocational	9; 27.27%	6; 20.69%	8; 25.81%	
Secondary	16; 48.48%	8; 27.59%	11; 35.48%	
Higher Education	6; 18.18%	12; 41.38%	8; 25.81%	
**Marital Status—*n*; %**				
Single/Widowed/Divorced	8; 24.24%	9; 31.03%	7; 22.58%	0.731 ^b^
Married/Cohabiting	25; 75.76%	20; 68.97%	24; 77.42%	
**Residential Status—*n*; %**				
Village	12; 36.36%	7; 24.14%	12; 38.71%	0.439 ^b^
Town/City	21; 63.64%	22; 75.86%	19; 61.29%	
**Employment—*n*; %**				
Employed	14; 42.42%	6; 20.00%	12; 38.71%	0.185 ^b^
Unemployed	0; 0.00%	0; 0.00%	1; 3.23%	
Pensioner	19; 57.58%	24; 80.00%	18; 58.06%	
**Treatment approach** **—** ***n*** **; %**				
Monotherapy	3; 9.09%	4; 13.33%	0; 0.00%	0.007 *^b^
Combination therapy	25; 75.76%	26; 86.67%	31; 100.00%	
Observation	5; 15.15%	0; 0.00%	0; 0.00%	
**Duration of Treatment—**				
months; median (IQR)	3; 6.00	8; 28.00	7;18.00	0.393 ^a^
**Line of Therapy—*n*; %**				
Watch-and-wait	5; 15.15%	0; 0.00%	0; 0.00%	0.007 *^b^
First Line of Therapy	28; 84.85%%	30; 100.00%	31; 100.00%	
**ECOG Performance Status** **—** ***n*** **; %**				
0	14; 42.42%	5; 16.67%	5; 16.13%	0.089 ^b^
1	17; 51.52%	21; 70.00%	25; 80.65%	
2	2; 6.06%	3; 10.00%	1; 3.23%	
3	-	1; 3.33%	-	
4	-	-	-	
5	-	-	-	
**Comorbidities** **—** ***n*** **; %**				
No Comorbidities	16; 48.48%	14; 46.67%	10; 32.26%	0.332 ^b^
Hypertension	11; 33.33%	12; 40.00%	15; 48.39%	
Hypertension and diabetes	3; 9.09%	3; 10.00%	6; 19.35%	
Hypothyroidism	2; 6.06%	0; 0.00%	0; 0.00%	
Immunodeficiency	1; 3.03%	0; 0.00%	0; 0.00%	
Rheumatoid arthritis	0; 0.00%	1; 3.33%	0; 0.00%	

Notes: ECOG, Eastern Cooperative Oncology Group; IQR, interquartile range; BMI, body mass index. * *p* < 0.05; ᵃ Kruskal–Wallis test; ᵇ chi-square test.

**Table 2:** Assessment results for quality of life, anxiety severity, coping strategies, and dominant-hand grip strength by study group.

Variable	Leukemia (*n* = 33) Me (IQR)	Myeloma (*n* = 30) Me (IQR)	Lymphoma (*n* = 31) Me (IQR)	H (df = 2)	*p*	p FDR-BH	ε^2^
	**EORTC QLQ-C30**						
QL2	66.66 25.00	58.33 (16.67)	58.33 (16.67)	5.71	0.057	0.290	0.040
PF2	80.00 (13.33)	73.33 (20.00)	80.00 (20.00)	3.95	0.139	0.474	0.021
RF2	83.33 (33.33)	91.66 (16.67)	83.33 (33.33)	0.39	0.822	0.858	0.000
EF	91.66 (33.33)	83.33 (16.67)	83.33 (25.00)	1.74	0.419	0.596	0.000
CF	83.33 (16.67)	83.33 (16.67)	100.00 (33.33)	0.25	0.880	0.880	0.000
SF	83.33 (33.33)	66.66 (33.3)	83.33 (33.33)	0.79	0.672	0.733	0.000
FA	33.33 (11.11)	33.33 (11.11)	33.33 (22.22)	2.04	0.359	0.576	0.000
NV	0.00 (16.67)	0.00 (00.0)	0.00 (00.0)	0.91	0.635	0.726	0.000
PA	16.66 (33.33)	16.66 (33.33)	33.33 (50.00)	2.73	0.255	0.542	0.008
DY	0.00 (33.33)	0.00 (0.00)	0.00 (33.33)	1.48	0.477	0.603	0.005
SL	33.33 (0.00)	33.33 (33.33)	33.33 (33.33)	1.72	0.422	0.596	0.003
AP	0.00 (33.33)	0.00 (33.33)	0.00 (0.00)	1.61	0.447	0.596	0.000
CO	0.00 (33.33)	0.00 (33.33)	0.00 (0.00)	3.69	0.158	0.474	0.018
DI	0.00 (33.33)	0.00 (0.00)	0.00 (0.00)	2.63	0.268	0.542	0.001
FI	0.00 (33.33) ^a^	0.00 (0.00) ^b^	0.00 (33.33) ^a^	9.03	0.010 *	0.087	0.077
	**STAI**						
X1	33.00 (13.00)	36.00 (11.00)	39.00 (15.00)	5.19	0.07	0.298	0.035
X2	40.00 (9.00) ^a^	48.00 (16.00) ^b^	50.00 (13.00) b	16.77	0.0002 *	0.003 *	0.162
	**Mini-MAC**						
HH	10.00 (6.00)	11.00 (4.00)	10.00 (5.00)	1.29	0.524	0.630	0.000
AP^1^	12.00 (8.00)	14.00 (6.00)	17.00 (9.00)	5.61	0.060	0.290	0.039
FS	24.00 (4.00)	23.00 (4.00)	23.00 (4.00)	2.35	0.309	0.562	0.003
PR	18.00 (2.00) ^a^	21.00 (3.00) ^b^	22.00 (6.00) ^b^	17.73	0.0001 *	0.003 *	0.173
K style	43.00 (7.00)	44.00 (4.00)	44.00 (8.00)	2.23	0.327	0.562	0.002
D style	20.00 (10.00)	24.50 (9.00)	27.00 (14.00)	2.93	0.231	0.542	0.010
**HGS**	33.50 (17.00)	25.50 (18.50)	29.00 (16.00)	2.61	0.27	0.542	0.006

Notes: EORTC QLQ-C30, European Organisation for Research and Treatment of Cancer Quality of Life Questionnaire-C30; QL2, global health status and quality-of-life scale; PF2, physical functioning scale; RF2, role functioning scale; EF, emotional functioning scale; CF, cognitive functioning scale; SF, social functioning scale; FA, fatigue scale; NV, nausea and vomiting scale; PA, pain scale; DY, dyspnea scale; SL, insomnia scale; AP, appetite loss scale from the EORTC QLQ-C30; CO, constipation scale; DI, diarrhea scale; FI, financial difficulties scale; STAI, State-Trait Anxiety Inventory; X1, state anxiety; X2, trait anxiety; Mini-MAC, Mini-Mental Adjustment to Cancer scale; HH, helplessness–hopelessness; AP^1^, anxious preoccupation from Mini-MAC; FS, fighting spirit; PR, positive reappraisal/redefinition; K style, constructive adjustment style; D style, destructive adjustment style; HGS, handgrip strength; Me, median; IQR, interquartile range; H, Kruskal–Wallis test statistic; df, degrees of freedom; *p*, *p*-value; ε^2^, epsilon-squared effect size. Letters (a, b) indicate groups that differed significantly in the multiple-comparison analysis, *p* < 0.05. Groups marked with the same letter did not differ significantly. Statistically significant results are marked with an asterisk (*).

**Table 3:** Linear regression model explaining variability in quality-of-life scale 2.

	Model: Dependent Variable: QL2							
Variable	M1 β	*p*	M2 β	*p*	M3 β	M3 B	M3 95%CI	*p*
Age	−0.177	0.06	−0.221	0.024 *	−0.119	−0.158	[−0.430, 0.114]	0.251
Sex	0.002	0.98	0.020	0.816	0.242	8.460	[−0.592, 17.511]	0.066
Treatment Duration	0.123	0.183	0.089	0.307	0.080	0.059	[−0.066, 0.184]	0.349
Myeloma vs. Leukemia	−0.227	0.038 *	−0.148	0.211	−0.129	−4.884	[−13.512, 3.743]	0.263
Lymphoma vs. Leukemia	−0.201	0.055	−0.159	0.177	−0.144	−5.335	[−13.748, 3.078]	0.210
Finances	−0.491	<0.001 *	−0.404	<0.001 *	−0.329	−0.242	[−0.388, −0.096]	0.001 *
STAI-X2	-	-	−0.304	0.002 *	−0.248	−0.446	[−0.807, −0.084]	0.016 *
PR	-	-	0.169	0.119	0.202	1.189	[−0.051, 2.429]	0.059
HGS	-	-	-	-	0.284	0.429	[0.010, 0.849]	0.045 *
Fatigue	-	-	-	-	−0.158	−0.121	[−0.279, 0.037]	0.130
R^2^	0.289		0.386		0.431			
Adjusted R^2^	0.240		0.328		0.361			
ΔR^2^	–		0.097		0.045			
F	5.843		6.628		6.212			
p Model	<0.001 *		<0.001 *		<0.001 *			
VIF max	1.42		1.90		2.82			
p Shapiro–Wilk test	0.518		0.384		0.286			
p Breusch Pagan test	0.873		0.976		0.943			
p RESET test	0.252		0.828		0.342			
Cook > 4/n	6		8		7			
Studentized residuals (|t| > 3)	1		1		1			

Notes: STAI-X2, State-Trait Anxiety Inventory, Trait Anxiety subscale; PR, positive reappraisal/redefinition; HGS, handgrip strength; R^2^, coefficient of determination; adjusted R^2^, adjusted coefficient of determination; ΔR^2^, change in the coefficient of determination; F, F-statistic; p model, *p*-value for the overall regression model; M1–M3, regression models 1–3; β, standardized regression coefficient; B, unstandardized regression coefficient; CI, confidence interval; *p*, statistical significance level (*p*-value); VIF max, maximum variance inflation factor; Cook > 4/n, Observations with Cook’s D > 4/n; Studentized residuals (|t| > 3), Observations with |studentized residual| > 3. Statistically significant results are marked with an asterisk (*).

**Table 4:** Multicollinearity diagnostics.

Model	VIF Sex	VIF Age	VIF HGS	Highest VIF
Model 2	1.111	1.287	—	1.896
Model 2 + HGS	2.386	1.475	2.817	2.817
Model 3	2.453	1.532	2.818	2.818

Notes: VIF, Variance Inflation Factor; HGS, handgrip strength.

## Data Availability

The data are available upon reasonable request from the corresponding author.

## Supplementary Materials

The following supporting information can be downloaded at https://www.mdpi.com/article/10.3390/jcm15218545/s1. Table S1. Unstandardized regression coefficients and 95% confidence intervals for the final multiple linear regression model.
