# Supplementary material for: Quality of Life Among Patients with Hematologic Malignancies Receiving Outpatient Care: Associations with Socioeconomic, Clinical, Psychological, and Physical Factors
Source: J Clin Med. 2026 Nov 3;15(21):8545. doi: 10.3390/jcm15218545 (PMC13764063; doi:10.3390/jcm15218545)
Supplement: Supplementary file 1 [file jcm-15-08545-s001.zip › jcm-4620751-supplementary.pdf]

Supplementary Table S1. Unstandardized regression coefficients and 95% confidence intervals for the final multiple linear regression model

**Predictor**	**B**	**95% CI (OLS)**	**95% CI (bootstrap)**
Age	−0.158	[−0.430, 0.114]	[-0.400; 0.085]
Sex	8.460	[−0.592, 17.511]	[-1.184; 16.963]
Treatment duration	0.059	[−0.066, 0.184]	[-0.081; 0.163]
Myeloma vs. leukemia	−4.884	[−13.512, 3.743]	[-12.749; 3.684]
Lymphoma vs. leukemia	−5.335	[−13.748, 3.078]	[-14.486; 3.950]
Finances	−0.242	[−0.388, −0.096]	[-0.373; -0.094]
STAI-X2	−0.446	[−0.807, −0.084]	[-0.783; -0.105]
PR	1.189	[−0.051, 2.429]	[0.025; 2.396]
HGS	0.429	[0.010, 0.849]	[-0.014; 0.816]
Fatigue	−0.121	[−0.279, 0.037]	[-0.312; 0.035]

Model performance and internal validation

**Statistic**	**Value**
Apparent R^2	0.431
Adjusted R^2	0.362
Mean bootstrap optimism in R^2	0.127
Optimism-corrected R^2	0.304
Successful bootstrap replications	2,000/2,000

Note. B, unstandardized regression coefficient; CI, confidence interval; OLS, ordinary least squares; R^2, coefficient of determination. Conventional OLS 95% confidence intervals were calculated using model-based standard errors and the t distribution, whereas bootstrap percentile 95% confidence intervals were derived from the empirical distributions of the bootstrap coefficient estimates using 2,000 bootstrap resamples. The reference category for diagnosis was leukemia. The optimism-corrected R^2 was calculated by subtracting the mean bootstrap optimism from the apparent R^2. The adjusted R^2 accounts for the number of predictors in the model.
